# Examining associations between genetic and neural risk for externalizing behaviors in adolescence and early adulthood

**DOI:** 10.1017/S0033291723001174

**Published:** 2023-05-19

**Authors:** Sarah J. Brislin, Jessica E. Salvatore, Jacquelyn M. Meyers, Chella Kamarajan, Martin H. Plawecki, Howard J. Edenberg, Samuel Kuperman, Jay Tischfield, Victor Hesselbrock, Andrey P. Anokhin, David B. Chorlian, Marc A. Schuckit, John I. Nurnberger, Lance Bauer, Gayathri Pandey, Ashwini K. Pandey, John R. Kramer, Grace Chan, Bernice Porjesz, COGA Collaborators, Danielle M. Dick

**Affiliations:** 1 Department of Psychiatry, Robert Wood Johnson Medical School, Rutgers University, New Brunswick-Piscataway, NJ, USA; 2 Department of Psychiatry and Behavioral Sciences, State University of New York Downstate Medical Center, New York, NY, USA; 3 Department of Psychiatry, Indiana University, Bloomington, IN, USA; 4 Department of Biochemistry and Molecular Biology, Indiana University, Bloomington, IN, USA; 5 Department of Psychiatry, University of Iowa, Iowa City, IA, USA; 6 Department of Psychiatry, University of Connecticut School of Medicine, Farmington, CT, USA; 7 Department of Psychiatry, Washington University in St. Louis, St. Louis, MO, USA; 8 Department of Psychiatry, University of California San Diego Medical School, San Diego, CA, USA

**Keywords:** Externalizing, neurophysiology, polygenic score, P3 amplitude

## Abstract

**Background.:**

Researchers have identified genetic and neural risk factors for externalizing behaviors. However, it has not yet been determined if genetic liability is conferred in part through associations with more proximal neurophysiological risk markers.

**Methods.:**

Participants from the Collaborative Study on the Genetics of Alcoholism, a large, family-based study of alcohol use disorders were genotyped and polygenic scores for externalizing (EXT PGS) were calculated. Associations with target P3 amplitude from a visual oddball task (P3) and broad endorsement of externalizing behaviors (indexed via self-report of alcohol and cannabis use, and antisocial behavior) were assessed in participants of European (EA; *N* = 2851) and African ancestry (AA; *N* = 1402). Analyses were also stratified by age (adolescents, age 12–17 and young adults, age 18–32).

**Results.:**

The EXT PGS was significantly associated with higher levels of externalizing behaviors among EA adolescents and young adults as well as AA young adults. P3 was inversely associated with externalizing behaviors among EA young adults. EXT PGS was not significantly associated with P3 amplitude and therefore, there was no evidence that P3 amplitude indirectly accounted for the association between EXT PGS and externalizing behaviors.

**Conclusions.:**

Both the EXT PGS and P3 amplitude were significantly associated with externalizing behaviors among EA young adults. However, these associations with externalizing behaviors appear to be independent of each other, suggesting that they may index different facets of externalizing.

Externalizing disorders (i.e. substance use disorders, antisocial behavior, attention-deficit/hyperactivity disorder [ADHD], oppositional defiant disorder [ODD], and conduct disorder [CD]) are highly comorbid (Krueger, Markon, Patrick, Benning, & Kramer, 2007; Krueger et al., 2021). There is robust evidence that the association across these disorders is explained, in part, through common neural and genetic processes (Karlsson Linnér et al., 2021; Kotov et al., 2017; Krueger et al., 2021; Patrick et al., 2006; Venables et al., 2017). Neurophysiological and genome-wide association studies (GWAS) are each powerful methods that have been used, mostly separately, to improve our understanding of externalizing disorder etiology. However, it is still unclear how genetic liability relates to the underlying neural mechanisms of these conditions. Separately, numerous studies have found that neural mechanisms relevant to controlling one’s ability to resist impulsive urges (i.e. executive control) develop over the course of adolescence and young adulthood (Shulman et al., 2016). Therefore, the current study seeks to leverage information from genetic and neurophysiological domains to improve our understanding of the biological bases of externalizing behaviors, and also to determine if genetic and neurophysiological liability shows differential associations with externalizing behavior in adolescence and early adulthood.

## Genetic liability for externalizing behaviors

The co-morbidity of externalizing disorders is largely attributed to shared genetic liability (Dick, Viken, Kaprio, Pulkkinen, & Rose, 2005; Waldman, Poore, van Hulle, Rathouz, & Lahey, 2016) and the heritability of a general externalizing factor has been estimated to be quite high (*h*^2^ 0.81–0.84; Krueger et al., 2007; Young, Stallings, Corley, Krauter, and Hewitt, 2000). GWAS of individuals of European ancestry (EA) have been recently used with great success to identify many genetic variants – typically single nucleotide polymorphisms (SNPs) – that are associated with complex traits including ADHD and alcohol use. Genomic structural equation modeling (gSEM) is a multivariate method developed for analyzing the joint genetic architecture of complex traits (Grotzinger et al., 2019), and was recently applied to data from GWAS of externalizing behaviors. This model used data from large GWAS (*N* > 50 000) available for seven externalizing phenotypes (ADHD, problematic alcohol use, lifetime cannabis use, age at first sexual intercourse, number of sexual partners, risk-taking, and lifetime smoking initiation). The model included data from 1.5 million EA individuals and indicated a single genetic factor underlying the externalizing behaviors (Karlsson Linnér et al., 2021), paralleling findings from twin data. More than 500 loci were associated with the externalizing factor at levels surpassing genome-wide significance. These loci were enriched for genes expressed in the brain and related to development of the nervous system (Karlsson Linnér et al., 2021).

Using the results from these analyses, polygenic scores (PGS) were calculated in samples not included in the GWAS, including from the Collaborative Study on the Genetics of Alcoholism (COGA) where it was found to explain 8.9% of the variance in a latent phenotypic factor among EA adults (Karlsson Linnér et al., 2021). The externalizing PGS (EXT PGS) was significantly associated with relevant externalizing phenotypes such as disinhibited behavior (e.g. rule breaking, aggression), externalizing disorders (e.g. ADHD, CD, alcohol use disorder), and related social outcomes including criminal justice involvement (e.g. arrest, felony conviction), and socioeconomic outcomes (e.g. lower levels of college completion, lower household income; Karlsson Linnér et al., 2021). While recent publications have continued to validate the EXT PGS, finding significant associations between the EXT PGS and externalizing outcomes among EA, but not African ancestry (AA) adolescents (Kuo et al., 2021), it is still unknown how the EXT PGS is associated with established neurophysiological indicators of externalizing.

## P3 amplitude and externalizing behaviors

Researchers have used event-related potentials and electroencephalography (EEG) to investigate biomarkers for psychiatric disorders for decades (Iacono, 2018). The P3 (also termed the P300) is a positivity in the scalp electrical potential that occurs between 300–700 ms following a ‘significant’ rare stimulus or ‘target’. The P3 is typically measured at central parietal electrodes where it is maximum and in this context is thought to reflect an estimate of effortful, ‘top down’ attentional shift. Twin studies indicate that the P3 is highly heritable (estimates ranging from 0.49–0.78; Katsanis, Iacono, McGue, & Carlson, 1997; O’Connor, Morzorati, Christian, & Li, 1994; Van Beijsterveldt, Molenaar, De Geus, & Boomsma, 1996). Low P3 amplitude derived from a visual oddball task is a well-documented neurophysiological index associated with a broad liability for externalizing psychopathology in adults and late adolescence, including substance and alcohol use disorders, ADHD, and antisocial behavior (Euser et al., 2012; Porjesz et al., 2005). Twin studies of primarily White samples have shown that the association between P3 amplitude and externalizing behaviors are due, in part, to genetic correlation (i.e. shared genetic influences; 4.8% of variance is shared) between these phenotypes (Gilmore, Malone, Bernat, & Iacono, 2010; Hicks et al., 2007; Yoon, Iacono, Malone, & McGue, 2006). Based on twin and family data, low P3 amplitude has been identified as a candidate endophenotype for externalizing behaviors (Iacono & Malone, 2011; Porjesz et al., 2005), suggesting that the neurophysiological characteristics of which P3 amplitude is a marker may mediate genetic liability. However, this evidence is based on the estimation of latent genetic factors. To our knowledge, no studies have employed *measured* genetic liability to provide direct tests of the hypothesis that P3 amplitude mediates the association between genetic predispositions and externalizing behaviors.

## Current study

The current pre-registered study focused on disentangling the relationship between genetic and one neural correlate of externalizing behaviors, P3 amplitude from a visual oddball task. This work is informed by the Hierarchical Taxonomy of Psychopathology (HiTOP) model (Kotov et al., 2017), which posits that the comorbidity seen across externalizing disorders can be accounted for by a general liability for externalizing behaviors. The HiTOP model takes an empirically-based approach toward refining our understanding of psychiatric symptoms and proposes that this approach may provide better targets for behavioral genetic and neural research than diagnoses, which have historically been hindered by heterogeneity and reduced reliability, validity, and statistical power (Kotov et al., 2017; Markon, Chmielewski, & Miller, 2011; Perkins, Latzman, & Patrick, 2020).

To our knowledge, only one recent study has examined associations between brain-based variables and PGS for alcohol use, cannabis use, smoking, schizophrenia, and educational attainment. This study found significant associations between brain-based variables and PGS for specific behaviors (e.g. regular smoking) through using a principal component analyses of multivariate EEG indicators (including P3 amplitude) instead of examining individual EEG indicators (Harper et al., 2021). In contrast, the current study focused specifically on externalizing behaviors as a phenotype of interest. Research on the genetic architecture of externalizing by Karlsson Linnér et al. (2021) as well as the longstanding literature linking the P3 amplitude from a visual oddball task to a broad phenotypic externalizing factor (Gilmore et al., 2010; Patrick et al., 2006) suggests that both the EXT PGS and P3 amplitude are ideal candidate indicators of a broad externalizing liability in their respective domains of measurement.

The current study sought to determine the associations between known genetic and neural risk indicators for externalizing behaviors to advance a biologically informed understanding of the etiology of externalizing behaviors. To do this, we used cross-sectional genetic, neurophysiological, and interview data from COGA from individuals ages 12 to 32.

Given prior findings, we hypothesized:

1. EXT PGS scores would be significantly and positively associated with increased externalizing behaviors in both adolescence and young adulthood (Karlsson Linnér et al., 2021; Kuo et al., 2021).
2. P3 amplitude would be significantly and negatively associated with increased externalizing behaviors in both adolescence and young adulthood (Porjesz et al., 2005).
3. EXT PGS scores would be significantly and negatively associated with P3 amplitude.
4. There would be an indirect effect of P3 amplitude on the association between EXT PGS and externalizing behavior, with the hypothesis that P3 amplitude would partially account for the variance shared between the EXT PGS and externalizing behaviors.

## Methods

### Sample

Data were from the COGA study (Edenberg, 2002). COGA is a diverse, multi-site, multi-generational, family-based study of genetic and environmental factors for alcohol use disorders (Begleiter et al., 1995, Reich et al., 1998). Families with multiple members with alcohol use disorders and community-based comparison families were recruited into the study and have been followed for over 30 years. The Institutional Review Board at all sites approved this study and written consent/assent was obtained from all participants. The present study includes all data available (originally recruited family members and offspring from the original COGA study, the COGA prospective study, and the COGA Interactive Research Project Grant study) for individuals ages 12 to 32 who met the following criteria (1) had GWAS data available, (2) completed the adolescent or adult Semi-Structured Assessment for the Genetics of Alcoholism (SSAGA) Interview (Bucholz et al., 1994), and (3) had electrophysiological data available collected at the time of a complete interview. This resulted in a total sample of 2851 EA individuals and 1402 AA individuals. Sample descriptions are included in Table 1.

### Measures

#### Phenotypic data

##### Externalizing Behavior Score:

Analyses used self-report data collected at the same experimental timepoint as EEG data.

Indicators differed between adolescents and young adults due to developmental differences in substance use and externalizing behaviors (e.g. low incidence rate of AUD symptom endorsement among adolescents), and the use of different assessments in COGA based on age. For adolescents, indicators included: alcohol use, cannabis use, and DSM-5 (American Psychiatric Association, 2013) symptom counts of CD and ODD. All indicators for adolescents were obtained from the Child Semi-Structured Assessment for the Genetics of Alcoholism (C-SSAGA), an interview for children and adolescents based on the SSAGA and developed for COGA (Bucholz et al., 1994). Alcohol use was determined in the C-SSAGA by asking individuals to report frequency of past 12 months drinking on a 12-point scale. The scale was reversed from the original coding such that in the current study 1 indicated the lowest level of drinking (about 1 to 2 days) and 12 indicated the maximum level of drinking (every day). Non-drinkers were coded as zero. Cannabis use was coded as 1 (any use in the past year) or 0 (no cannabis use use).

For young adults, all indicators were measured using the SSAGA, which has been found to produce reliable and valid DSM-based criterion counts (Bucholz et al., 1994, Hesselbrock, Easton, Bucholz, Schuckit, & Hesselbrock, 1999). For young adults, indicators were: number of DSM-5 (American Psychiatric Association, 2013) alcohol use disorder symptoms endorsed, number of cannabis use disorder symptoms endorsed, number of adult antisocial behavior symptoms, and number of CD symptoms.

Confirmatory factor analyses were performed in MPlus (version 8; (Muthen & Muthén, 1998–2017) to estimate factor scores for each group (EA adolescents, EA young adults, AA adolescents, AA young adults) using the weighted least square mean and variance adjusted (WLSMV) estimator for adolescents and robust maximum likelihood (MLR) estimator for young adults. WLSMV is a robust estimator that does not assume normally distributed variables and provides the best option for modeling a mix of categorical and continuous variables (Li, 2016). MLR is a robust estimator that also does not assume normally distributed variables and is the best option for continuous variables (Li, 2016). Factor models were specified by fixing latent factor means to 0 and variances to 1. Among the phenotypic indicators, 2.04% of data was missing for adolescents (1.69% for EA, 2.71% for AA) and 0.45% of data was missing for young adults (0.34% EA, 0.68% AA). For both adolescents (CFI: 0.865; RMSEA: 0.126, 95% CI 0.109–0.143; SRMSR: 0.106) and young adults (CFI: 0.884; RMSEA: 0.105, 95% CI 0.092–0.118; SRMSR: 0.081) the model fit statistics fell close to, but just outside of the commonly reported thresholds for a ‘good’ fitting model. Item descriptions and model details, including factor loading and model fit statistics are included in online Supplementary Table 1.

#### Genetic data

DNA samples were genotyped using the Illumina Human1 M array (Illumina, San Diego, CA), The Illumina Human OmniExpress 12V1 array (Illumina), the Illumina 2.5 M array (Illumina) or the Smokescreen genotyping array (Biorealm LLC, Walnut, CA; Baurley, Edlund, Pardamean, Conti, & Bergen, 2016). Details of the data processing, quality control, and imputation are provided in detail elsewhere (Lai et al., 2019). Data were imputed to 1000 Genome Phase 3 and SNPs with a genotypic rate <0.95, that violated Hardy–Weinberg equilibrium ( *p* < 10^−6^) or had minor allele frequency <0.01 were excluded from analyses.

##### Externalizing polygenic scores (EXT PGS):

Genetic liability for externalizing problems was assessed by constructing PGS. Effect sizes from GWAS summary statistics from analyses by Karlsson Linnér et al. (2021) were used to aggregate and weight the risk alleles carried by each individual (see Karlsson Linnér et al., 2021 for additional details regarding the computation of the latent genetic externalizing factor).

The EXT PGS scores were calculated using PRS-CS (Ge, Chen, Ni, Feng, & Smoller, 2019). PRS-CS uses a Bayesian regression and continuous shrinkage method to correct for the non-independence among nearby SNPs. Per recommendations of the PRS-CS developers, SNPs in the EXT PGS were limited to those from HapMap3 that overlapped between the original GWAS summary statistics and the LD reference panel (1000 Genomes Phase III reference panel). For participants of EA, we used estimates from Karlsson Linnér et al. (2021) to compute the EXT PGS scores. For individuals of AA, the EXT PGS was constructed using the weights from the results of the GWAS based on EA samples, noting that summary statistics from an ancestry matched GWAS are not currently available. EXT PGS scores were standardized (z-scored) to improve the interpretability of results.

#### Neurophysiological data

##### P3 amplitude:

Stimuli and methods of data collection and processing for event related potentials have been described in previous studies of the Visual Oddball Paradigm in COGA (Cohen et al., 1994; Porjesz & Begleiter, 1998). Consistent with previous studies using COGA data, the current study examined peak amplitude, relative to the pre-stimulus baseline, of P3 to target stimuli in the 250–600 ms time window at the Pz (midline parietal) electrode. This task and task parameters were chosen due to the large, existing body of literature linking P3 response under these conditions and externalizing liability (Gilmore et al., 2010; Iacono & Malone, 2011; Porjesz et al., 2005). If individuals had full data including P3 data from more than one timepoint, the P3 amplitude from their last (oldest age) data collection and matched self-report data were used.

#### Analytic plan

This study followed a preregistered analysis plan (https://osf.io/4f5x8). The regression analyses were cross-sectional and conducted in R (R Core Team, 2021). First, to test Hypothesis 1, the externalizing behavior score was regressed on the EXT PGS. To test Hypothesis 2, the externalizing behavior score was regressed on P3 amplitude. Hypothesis 3 was tested by regressing P3 amplitude on EXT PGS. We then performed mediation analyses (Hypothesis 4) to determine the indirect effect of P3 amplitude on the association between EXT PGS (independent variable) and the externalizing behavior score (dependent variable). All analyses included relevant covariates as applicable (top 10 ancestry principal components, age, sex, etc.).

As COGA is a family-based study, cluster corrected standard errors were computed to account for the non-independence of these observations. All analyses were stratified by ancestry and age group, such that associations for EA adolescents (12–17 years old), EA young adults (18–31 years old), AA adolescents, and AA young adults were all analyzed separately. As epidemiologic (Eme, 2016) and neurophysiological studies (Iacono, Malone, & McGue, 2003; Porjesz & Begleiter, 1998) have found sex differences such that males endorse higher levels of externalizing behaviors and have smaller P3 amplitudes in comparison to females, sex was included as a covariate in all analyses. Follow up analyses including the interactive effects of sex were also performed. To test interactive effects relevant to Hypothesis 1 and 3, EXT PGS by sex, EXT PGS by age, and age by sex interaction terms were added to the base models (Keller, 2014). To test the interactive effects relevant to Hypothesis 2, the sex by P3 interaction term was added to the base model.

## Results

### Hypothesis 1: associations between EXT PGS and externalizing behaviors

#### Adolescents

**Figure 1.**
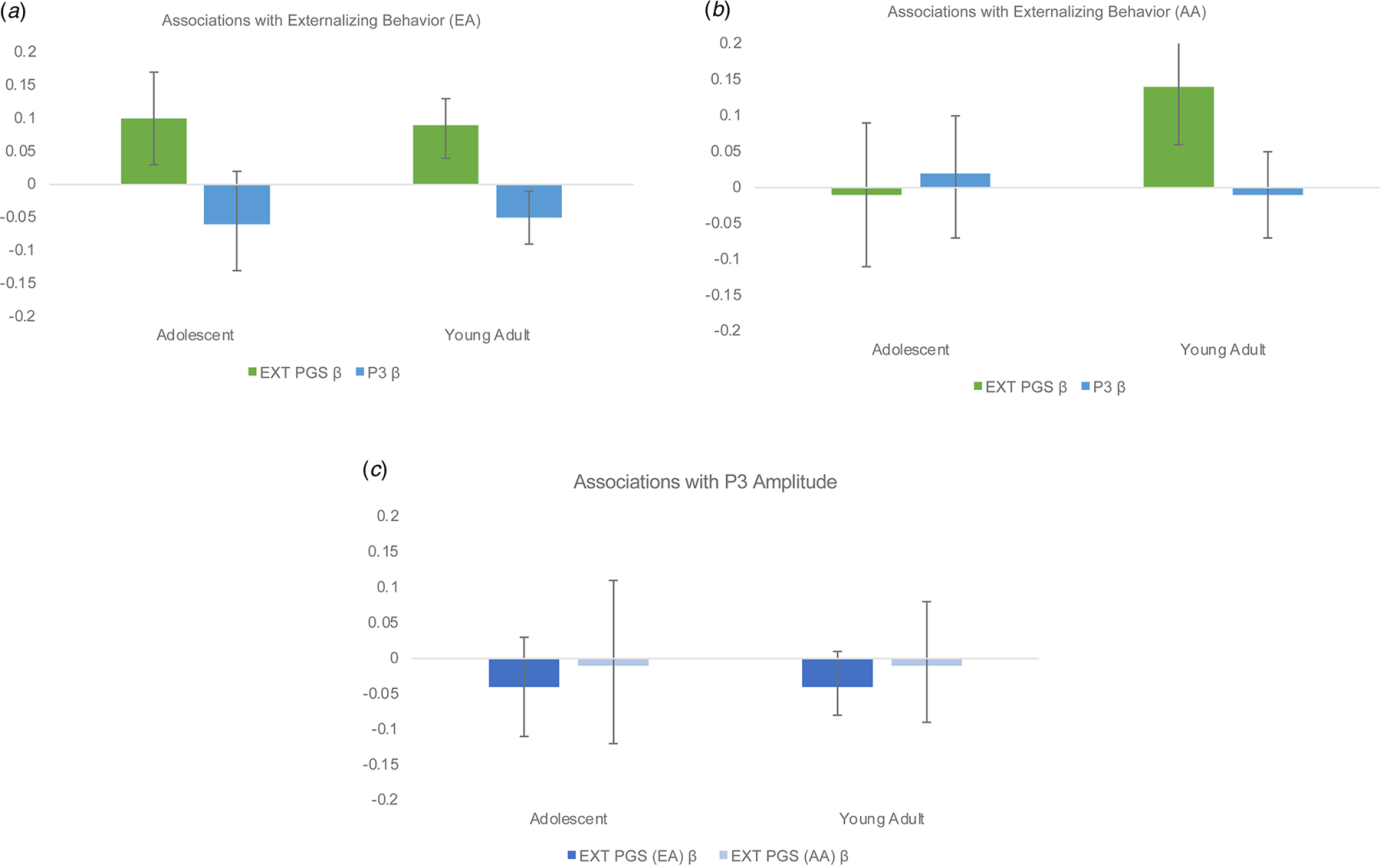
Associations among study variables. (*a*) Associations with externalizing behavior score among EA individuals. *β* are standardized beta weights from separate linear regression models for EXT polygenic score (PGS) and Visual oddball P3 amplitude (P3). (*b*) Associations with externalizing behavior score among AA individuals. (*c*) Association between P3 amplitude and EXT PGS scores for European (EA) and African (AA) ancestry individuals. *β* are standardized beta weights from separate linear regression models for EXT PGS and Visual oddball P3 amplitude (P3).

There was a significant association between the EXT PGS and externalizing behaviors among EA adolescents (*β*_EA_ = 0.10, 95% CI 0.03–0.17; Δ*R*^2^ = 0.01, 95% CI 0.00–0.02; Table 2, Fig. 1a), such that individuals who scored higher on the EXT PGS also reported higher levels of externalizing behaviors. There was also a significant effect of sex (*β* = −0.09, 95% CI −0.16 to −0.02; Table 2), indicating that males endorsed higher levels of externalizing behaviors. However, when tested, there was no evidence of a significant EXT PGS by sex interaction (*β* = −0.01, 95% CI −0.07 to 0.07). The association between the EXT PGS and externalizing behavior scores was not significant for AA adolescents (*β* = −0.01, 95% CI −0.10 to 0.09; Δ*R*^2^ = 0.00, 95% CI −0.00 to 0.00; Table 2, Fig. 1b); however, there was a similar effect of sex, such that males endorsed higher levels of externalizing behaviors (*β* = −0.11, 95% CI −0.20 to −0.03; Table 2). When tested, there was no evidence of a significant EXT PGS by sex interaction among EA or AA adolescents (online Supplementary Table 3).

#### Young adults

For EA and AA young adults there were significant associations between EXT PGS and externalizing behavior scores (*β*_EA_ = 0.08, 95% CI 0.03 to 0.12; $\Delta R_{\text{EA}}^{2}=0.01$, 95% CI 0.00 to 0.01; *β*_AA_ = 0.13, 95% CI 0.05 to 0.22; $\Delta R_{\text{AA}}^{2}=0.01$, 95% CI 0.00 to 0.03; Table 2, Fig. 1a, b)^†1^ as well as significant main effects of sex (*β*_EA_ = −0.26, 95% CI −0.30 to −0.22; *β*_AA_ = −0.31, 95% CI −0.38 to −0.25). When the interactive effect of EXT PGS and sex was included in the linear regression models, there were significant improvements in the model for EA (*β* = −0.05, 95% CI −0.10 to −0.01) but not AA young adults (*β* = −0.05, 95% CI −0.14 to 0.01; online Supplementary Table 3). These results indicate that among young adults, the association between EXT PGS and externalizing behaviors was strongest for males.

### Hypothesis 2: associations between P3 and externalizing behaviors

#### Adolescents

For EA adolescents, at the bivariate level, P3 amplitude was significantly associated with externalizing behavior scores (*r* = −0.07). However, in a regression model including age and sex as covariates, the association did not maintain significance (*B*_EA_ = −0.01, *β* = −0.06, 95% CI −0.13 to 0.02, Δ*R*^2^ = 0.00, 95% CI −0.00 to 0.01; Fig. 1a). At the bivariate level, as well as within the regression model, there were no significant associations between P3 amplitude and externalizing behavior scores for AA adolescents (*r* = 0.00; *B*_AA_ = 0.00, *β* = 0.02, 95% CI −0.07 to 0.10, Δ*R*^2^ = 0.00, 95% CI −0.00 to 0.00; Table 2, Fig. 1b). For EA and AA adolescents there were significant main effects of sex such that males endorsed higher levels of externalizing behaviors (*β*_EA_ = −0.11, 95% CI −0.18 to −0.05; *β*_AA_ = −0.12, 95% CI −0.21 to −0.03). When tested, there was no evidence of a significant EXT PGS by sex interactions (*β*_EA_ = −0.02, 95% CI −0.10 to 0.06; *β*_AA_ = −0.02, 95% CI −0.11 to 0.06; online Supplementary Table 3).

#### Young adults

The bivariate association between P3 amplitude and externalizing behavior among EA and AA young adults was significant (*r*_EA_ = −0.09; *r*_AA_ = −0.08). In the regression model including age and sex as covariates, P3 amplitude maintained the significant association with externalizing behavior among EA (*B*_EA_ = −0.01, *β*_EA_ = −0.05, 95% CI −0.09 to −0.01, Δ*R*^2^ = 0.002, 95% CI 0.00–0.01) but not AA individuals (*B*_AA_ = 0.00, *β*_AA_ = −0.01, 95% CI −0.07 to 0.05, Δ*R*^2^ = 0.00, 95% CI −0.00 to 0.00; Table 2, Fig. 1a, b). As previously reported, there was a significant main effect of sex (*β*_EA_ = −0.26, 95% CI −0.30 to −0.21; *β*_AA_ = −0.31, 95% CI −0.38 to −0.25), such that males scored higher on externalizing behavior; however, both P3 by sex interactions were non-significant (*β*_EA_ = 0.02, 95% CI −0.02 to 0.06; *β*_AA_ = 0.05, 95% CI −0.01 to 0.11; online Supplementary Table 3).

### Hypothesis 3: associations between EXT PGS and P3

#### Adolescents

Among EA and AA adolescents, the EXT PGS was not significantly associated with P3 amplitude (*β*_EA_ = −0.04, 95% CI −0.11 to 0.03; *β*_AA_ = −0.01, 95% CI −0.12 to 0.11; Table 3, Fig. 1c). There was not a significant main effect of sex for either ancestry group (*β*_EA_ = 0.04, 95% CI −0.03 to 0.11; *β*_AA_ = 0.05, 95% CI −0.05 to 0.14; Table 3), nor was there a significant sex by EXT PGS interaction (*β*_EA_ = −0.04, 95% CI −0.11 to 0.03; *β*_AA_ = −0.07, 95% CI −0.18 to 0.01; online Supplementary Table 4)

#### Young adults

Among EA and AA young adults, the EXT PGS was not significantly associated with P3 amplitude (*β*_EA_ = −0.04, 95% CI −0.11 to 0.03; *β*_AA_ = −0.01, 95% CI −0.09 to 0.08; Table 3, Fig. 1c)^2^. For both EA and AA young adults, sex was significantly associated with P3 amplitude such that P3 amplitude was higher among females (*β*_EA_ = 0.09, 95% CI 0.05–0.13; *β*_AA_ = 0.17, 95% CI 0.11–0.24; Table 3); however the EXT PGS by sex interaction term was not significant for EA or AA young adults (*β*_EA_ = −0.02, 95% CI −0.07 to 0.02; *β*_AA_ = −0.03, 95% CI −0.13 to 0.05; online Supplementary Table 4).

### Hypothesis 4: mediation analyses

#### Adolescents and young adults

The EXT PGS was not significantly associated with P3 amplitude. Consistent with this finding, the indirect effects of P3 amplitude on the association between the EXT PGS and externalizing behavior from all mediation models were not significant. When the externalizing behavior score was simultaneously regressed on the EXT PGS and P3 amplitude (and relevant covariates), the magnitude of association was similar to the separate models for both the EXT PGS and P3 amplitude (Table 4). For example, when the externalizing score was regressed on the EXT PGS and P3 amplitude in EA young adults, both independent variables remained significantly associated with externalizing behavior (*β*_EXT PGS_ = 0.09 [95% CI 0.04–0.13], *β*_P3_ = −0.05, [95%CI −0.09 to −0.01]), with standardized beta values of the same magnitude as those reported in Table 3, when each independent variable was modeled separately. Therefore, the EXT PGS and P3 amplitude account for unique and independent variation in externalizing behavior in this sample.

## Discussion

The current study sought to determine the associations between known genetic contributors and a specific neural risk factor, the visual oddball task related target P3, for a broad liability for externalizing behaviors within adolescents and young adults. We found support for Hypothesis 1, that the EXT PGS was positively associated with the externalizing behavior score in young adults and EA adolescents. Hypothesis 2 was partially supported such that blunted P3 amplitude was associated with increased externalizing behavior scores; however, this was only significant among EA young adults. Hypothesis 3 – that higher EXT PGS would be associated with lower P3 amplitude – was also supported, but again, only among EA young adults. Lastly, we did not find evidence that P3 amplitude accounted for the association between the EXT PGS and externalizing behavior (Hypothesis 4). P3 amplitude was not significantly associated with the EXT PGS and the two variables were statistically independent in analyses where they were both included in the same regression model. The present study adds to the literature in advancing the understanding of the mechanisms through which genetic liability is, and is not, conferred for externalizing behaviors.

The current study supports previous findings that both the EXT PGS and P3 amplitude are significantly associated with externalizing behaviors in COGA, as well as in other samples (Iacono, Malone, & Vrieze, 2017; Karlsson Linnér et al., 2021; Kuo et al., 2021; Porjesz et al., 2005). Findings from the current study are consistent with previous COGA findings that the EXT PGS was significantly associated with an externalizing behavior factor among EA, but not AA, adolescents (Kuo et al., 2021). The association between the EXT PGS and externalizing behavior among EA young adults is consistent with findings from the original paper describing the multivariate GWAS (Karlsson Linnér et al., 2021), and extend the association by also finding a significant association between the EXT PGS and externalizing behavior among AA young adults. Due to the cross-sectional nature of the data, these analyses cannot directly speak to the impact of genetic liability across development; however, the significant associations between the EXT PGS and externalizing behavior among adolescents and young adults suggest that the EXT PGS impacts the expression of externalizing behavior across a wide range of development.

The variables which comprise the externalizing factors differed between adolescence and young adulthood. The variables used in young adulthood reflect problems and impairment related to substance use and externalizing behaviors (i.e. DSM symptom counts of AUD, CUD, CD, and ASPD). The variables from adolescence, however, reflect greater problems and impairment related to impulsive and rule breaking behaviors (i.e. ODD and CD symptoms) than endorsement of any cannabis use and frequency of alcohol use. As expected, the externalizing factors differ somewhat between adolescence and young adulthood, corresponding to expected developmental changes. Despite these differences in how the externalizing factor was defined, associations between the EXT PGS and externalizing behavior were relatively consistent. This suggests that the EXT PGS may confer risk for externalizing behaviors in part, due to a shared mechanistic process (i.e. liability for impaired behavioral control) that is expressed differentially across development. Future studies examining these associations longitudinally are needed to provide a more comprehensive understanding of how phenotypic expression of genetic liability unfolds.

Our results also suggest a nuanced interpretation of sex differences in externalizing liability. We found evidence for an EXT PGS by sex interaction among EA young adults. When probed, these results indicated that among young adults, the association between the EXT PGS and externalizing behaviors was strongest for males. Again, longitudinal data is needed to understand how sex impacts differences in phenotypic expression of genetic liability for externalizing across development.

The association between P3 amplitude and externalizing behavior was not significant among AA participants when accounting for age and sex effects. While the magnitude of association between P3 amplitude and externalizing behavior was similar at the bivariate level among both EA and AA young adults (*r*_EA_ = −0.09, *r*_AA_ = −0.08), in the regression models when age and sex covariates are included, the association was no longer significant for AA participants. This may be due to smaller *N*’s for the AA adolescent and young adult groups, making it more difficult to detect small effects. Further research is needed with larger, diverse samples to address this limitation. The association between P3 amplitude and externalizing behavior was also not significant among EA adolescent participants when accounting for age and sex effects. However, the magnitude of the association between P3 amplitude and externalizing behavior among EA adolescents (*β* = −0.06) was similar to the significant association seen in the EA young adult group (*β* = −0.05). Therefore, the lack of significance among EA adolescents may be due to limitations of sample size (the largest *N* was available for EA young adults).

These results contribute to an emerging field of research examining the association between PGS and brain-based indicators. Previous work in the area of schizophrenia has found null results when attempting to link genetic liability scores and EEG-based indicators (Liu et al., 2017). The results from the current study are somewhat consistent with findings from Harper et al. (2021), which found that genetic liability for substance use behaviors (drinks per week, regular smoking, cannabis use) was significantly associated with EEG-based indicators. However, the principal component defined in part by event-related P3 amplitude was not significantly associated with any substance use behavior PGS’s (Harper et al., 2021).

This is the first study to examine both biologically-based variables – P3 and the EXT PGS – concurrently to determine the indirect effect of P3 amplitude on the association between EXT PGS and externalizing behaviors. Our findings suggest that both known genetic (EXT PGS) and neurophysiological (P3 amplitude) risk markers each contribute independently to the expression of externalizing behaviors. However, we do not view these findings as a definitive disconfirmation of the hypothesis that P3 amplitude mediates the association between EXT PGS and externalizing behaviors. The data used in these analyses are all cross-sectional and therefore not suited to make causal inferences. Also, issues of measurement error and small sample size need to be considered in the interpretation of these results. Therefore, future research efforts in large, longitudinal datasets should attempt to further test any theoretical mediation models and analyses should be replicated as more powerful measures of genetic risk become available.

As this was an initial attempt to understand the association between the EXT PGS, P3, and externalizing behaviors, we took a cross-sectional approach to maximize the sample size. In the future, longitudinal data will be used to determine the developmental trajectories of both P3 and externalizing behaviors and the impact of genetic liability on both these trajectories. These results suggest that P3 and the EXT PGS each index different facets of externalizing liability. The EXT PGS is formed from GWAS of substance use and risk-taking behaviors but did not include GWAS for antisocial or aggressive behavior as there were not samples available with sufficient power (all *N* < 50 000). Similarly, P3 amplitude is just one index of neurophysiological functioning that is relevant to externalizing psychopathology. Therefore additional, relevant electrophysiological phenotypes (e.g. Error Related Negativity, event-related oscillations) should be evaluated as potential brain-based responses that may partially account for the association between the EXT PGS and externalizing outcomes.

These results should be interpreted in the context of the following limitations. First, these results are cross-sectional and therefore cannot speak to the impact of genetic and neural risk on externalizing behaviors over time. In addition, CFAs were used to capture variance shared across externalizing behaviors, creating Externalizing Behavior factor scores. The model fit statistics for theses CFAs were close to, but fell outside the range of a ‘good’ fitting model. The approach also results in factor scores that are specific to the sample in which they were created, decreasing the generalizability of this outcome. Second, while the overall COGA sample is relatively large and diverse, necessary stratification by age and ancestry resulted in some of the analyses being performed in relatively small subgroups. Therefore, it is important that these results be replicated in a larger sample where more complex models (e.g. moderation of association between P3 amplitude and externalizing behavior by the EXT PGS) can be tested. PGS are by nature imprecise as they are an aggregation of variants that are associated with specific behaviors or diagnoses and contain noise that can obscure the EXT PGS association with relevant measures in other domains, including P3 amplitude. In addition, the EXT PGS was derived from a multivariate GWAS that only included EA individuals, and the predictive performance of EA-derived PGS is lower in non-EA samples (Duncan et al., 2019). Lastly, environmental variables play a critical role in the development and expression of externalizing behaviors and future work should incorporate environmental covariates (e.g. education, parenting style) as they may buffer the associations between the EXT PGS, P3 amplitude, and externalizing behavior.

Despite these limitations, the results of the current study provide an important step toward characterizing the etiology of risk for externalizing psychopathology. Understanding how genetic, neural, and behavioral risk for externalizing fit together and the developmental periods during which these associations are strongest provides an important step toward understanding the mechanisms through which genetic liability impacts psychopathology.

## Supplementary Material

Supplementary Material

## Figures and Tables

**Table 1.** Sample descriptives

Variable	European ancestry (12–17.9 years old; *N* = 890)			African ancestry (12–17.9 years old, *N* = 480)		
	M/%	s.d./n	Min, Max	M/%	s.d./n	Min, Max
Age	15.86	1.36	12.02, 17.95	15.77	1.42	12.07, 17.85
Female	52.5%	467		53.1%	255	
P3 Amplitude (*μ*)	27.30	8.61	0.48, 52.47	23.00	8.51	−3.19, 52.70
Externalizing Score	0.00	1.32	−1.11, 6.66	0.00	0.94	−0.80, 4.56
Variable	European ancestry (18–32 years old; *N* = 1961)			African ancestry (18–32 years old, *N* = 922)		
	M/%	s.d./n	Min, Max	M/%	s.d./n	Min, Max
Age	24.34	3.84	18.01, 31.86	24.07	3.78	18.02–31.81
Female	52.0%	1020		53.5%	493	
P3 Amplitude (μ)	22.70	8.67	−14.01, 55.01	17.09	8.03	−2.56, 42.68
Externalizing Score	0.00	1.27	−1.12, 6.94	0.00	1.17	−1.29, 4.75

Note: M, mean; SD, standard deviation; P3, visual P3; *μ*: microvolts.

**Table 2.** Regression results using externalizing behavior score as the criterion

Predictor	European ancestry 12–18 years old				African ancestry 12–18 years old			
	
	*β*	[95% CI]	*r*	*Δ R^2^* [95% CI]	*β*	[95% CI]	*r*	*Δ R^2^* [95% CI]

EXT PGS	0.10*	[0.03–0.17]		0.011 [0.00–0.02]	−0.01	[−0.11 to 0.09]		0.00 [−0.00 to 0.00]
Age	0.20*	[0.15–0.26]	0.20**		0.25**	[0.17–0.32]	0.25**	
Sex	−0.11*	[−0.18 to −0.04]	−0.11**		−0.12**	[−0.21 to −0.03]	−0.11**	

P3 Amplitude	−0.06	[−0.13 to 0.02]	−0.07*	0.004 [−0.00 to 0.01]	0.02	[−0.07 to 0.10]	0.00	0.00 [−0.00 to 0.00]
Age	0.19**	[0.14–0.25]	0.20**		0.25**	[0.17–0.32]	0.25**	
Sex	−0.11**	[−0.18 to −0.05]	−0.11**		−0.12*	[−0.21 to −0.03]	−0.11**	

Predictor	European ancestry 18–32 years old				African ancestry 18–32 years old			
	
	*β*	[95% CI]	*r*	*Δ R^2^* [95% CI]	*β*	[95% CI]	*r*	*Δ R^2^* [95% CI]

EXT PGS	0.09**	[0.04–0.13]		0.007 [0.00–0.01]	0.14*	[0.06–0.23]		0.013 [0.00–0.03]
Age	0.11**	[0.06–0.16]	0.09**		0.08**	[0.03–0.14]	0.06	
Sex	−0.26**	[−0.30 to −0.22]	−0.26**		−0.32**	[−0.38 to −0.25]	−0.31**	

P3 Amplitude	−0.05*	[−0.09 to −0.01]	−0.09**	0.002 [0.00–0.01]	−0.01	[−0.07 to 0.05]	−0.08*	0.00 [−0.00 to 0.00]
Age	0.10**	[0.05–0.15]	0.09**		0.09**	[0.03–0.15]	0.06	
Sex	−0.26**	[−0.30 to −0.21]	−0.25**		−0.31**	[−0.38 to −0.25]	−0.31**	

*Note.* Externalizing behavior score derived from confirmatory factor analysis for adolescents (comprised of alcohol use, cannabis use, DSM-5 symptom counts of Conduct Disorder and Oppositional Defiant Disorder) and young adults (comprised of DSM-5 symptom counts of alcohol use disorder, cannabis use disorder, adult antisocial personality disorder, and conduct disorder). *β* indicates the standardized regression weights. *r* represents the zero-order correlation. 95% CI indicates the lower and upper limits of the 95% confidence interval, respectively. All models also include the top 10 ancestry components.

* indicates *p* < 0.05.

** indicates *p* < 0.01.

**Table 3.** Regression results using P3 amplitude as the criterion

Predictor	European ancestry 12–18 years old				African ancestry 12–18 years old			
	*β*	[95% CI]	*r*	*Δ R^2^* [95% CI]	*β*	[95% CI]	*r*	*Δ R^2^* [95% CI]
EXT PGS	−0.04	[−0.11 to 0.03]		0.001 [−0.00 to 0.01]	−0.01	[−0.12 to 0.11]		0.000 [−0.00 to 0.00]
Age	−0.06	[−0.13 to 0.00]	−0.07*		−0.04	[−0.14 to 0.06]	−0.04	
Sex	0.04	[−0.03 to 0.11]	0.04		0.05	[−0.05 to 0.14]	0.05	
Predictor	European ancestry 18–32 years old				African ancestry 18–32 years old			
	*β*	[95% CI]	*R*	*Δ R^2^* [95% CI]	*β*	[95% CI]	*r*	*Δ R^2^* [95% CI]
EXT PGS	−0.04	[−0.08 to 0.01]		0.001 [−0.00 to 0.00]	−0.01	[−0.09 to 0.08]		0.000 [−0.00 to 0.00]
Age	−0.24**	[−0.28 to −0.20]	−0.24**		−0.17**	[−0.23 to −0.10]	−0.17**	
Sex	0.09**	[0.05–0.13]	0.08**		0.17**	[0.11–0.24]	0.16**	

*Note. β* indicates the standardized regression weights. *r* represents the zero-order correlation. 95% CI indicates the lower and upper limits of the 95% confidence interval, respectively. All models also include the top 10 ancestry principal components.

* indicates *p* < 0.05.

** indicates *p* < 0.01.

**Table 4.** Regression results using externalizing behavior as the criterion variable

Predictor	European ancestry 12–18 years old			African ancestry 12–18 years old		
	*β*	[95% CI]	Model Fit [95% CI]	*β*	[95% CI]	Model Fit [95% CI]
EXT PGS	0.10**	[0.03–0.18]		0.00	[−0.11 to 0.10]	
P3 Amplitude	−0.07*	[−0.13 to 0.00]		0.01	[−0.07 to 0.09]	
Age	0.19	[0.13–0.25]		0.26**	[0.18–0.33]	
Sex	−0.10	[−0.17 to −0.04]		−0.12**	[−0.20 to −0.03]	
			*R^2^* = 0.090**			*R^2^* = 0.124**
			[0.05–0.11]			[0.05–0.16]
Predictor	European ancestry 18–32 years old			African ancestry 18–32 years old		
	*β*	[95% CI]	Model Fit [95% CI]	*β*	[95% CI]	Model Fit [95% CI]
EXT PGS	0.09**	[0.04–0.13]		0.14**	[0.07–0.22]	
P3 Amplitude	−0.05*	[−0.09 to −0.01]		−0.02*	[−0.08 to −0.05]	
Age	0.10**	[0.05–0.15]		0.08**	[0.02–0.14]	
Sex	−0.26	[−0.30 to −0.22]		−0.31	[−0.38 to 0.25]	
			*R^2^* = 0.094**			*R^2^* = 0.147**
			[0.06–0.11]			[0.10–0.18]

*Note.* Externalizing behavior score derived from confirmatory factor analysis for adolescents (comprised of alcohol use, cannabis use, DSM-5 symptom counts of Conduct Disorder and Oppositional Defiant Disorder) and young adults (comprised of DSM-5 symptom counts of alcohol use disorder, cannabis use disorder, adult antisocial personality disorder, and conduct disorder). *β* indicates the standardized regression weights. 95% CI indicates the lower and upper limits ofthe 95% confidence interval, respectively. All models also include the top 10 ancestry components.

* indicates *p* < 0.05.

** indicates *p* < 0.01.

## Footnotes

† The notes appear after the main text.

1. As a test of robustness, when level of education was included in regression models alongside the EXT PGS, the results do not change, with the EXT PGS significantly associated with the externalizing behavior score (*β*_EA_ = 0.06 [95% CI 0.02 to 0.10], *β*_AA_ = 0.11, [95%CI 0.03 to 0.18]).

2. The inclusion of level of education in the regression analyses alongside the EXT PGS did not change the association with P3 amplitude, which remained non-significant (*β*_EA_ = −0.03 [95% CI −0.07 to 0.01], *β*_AA_ = −0.01, [95%CI: −0.09 to 0.07]).

## References

[R1] American Psychiatric Association. (2013). Diagnostic and statistical manual of mental disorders: DSM-5^™^ (5th ed., pp. xliv, 947). Arlington, VA, US: American Psychiatric Publishing, Inc. 10.1176/appi.books.9780890425596.

[R2] Baurley JW, Edlund CK, Pardamean CI, Conti DV, & Bergen AW (2016). Smokescreen: a targeted genotyping array for addiction research. BMC Genomics, 17(1), 1–12. 26921259 10.1186/s12864-016-2495-7 PMC4769529

[R3] Begleiter H, Reich T, Hesselbrock V, Porjesz B, Li TK, Schuckit MA, … Rice JP (1995). The collaborative study on the genetics of alcoholism. Alcohol Health and Research World, 19, 228–236. 31798102 PMC6875768

[R4] Bucholz KK, Cadoret R, Cloninger CR, Dinwiddie SH, Hesselbrock VM, Nurnberger JI, … Schuckit MA (1994). A new, semi-structured psychiatric interview for use in genetic linkage studies: A report on the reliability of the SSAGA. Journal of Studies on Alcohol, 55(2), 149–158. https://doi.org/10/gn6gww. 8189735 10.15288/jsa.1994.55.149

[R5] Cohen HL, Wang W, Porjesz B, Bauer L, Kuperman S, O’Connor SJ, … Begleiter H (1994). Visual P300: an interlaboratory consistency study. Alcohol, 11(6), 583–587. 7865162 10.1016/0741-8329(94)90087-6

[R6] Dick DM, Viken RJ, Kaprio J, Pulkkinen L, & Rose RJ (2005). Understanding the covariation among childhood externalizing symptoms: Genetic and environmental influences on conduct disorder, attention deficit hyperactivity disorder, and oppositional defiant disorder symptoms. Journal of Abnormal Child Psychology, 33(2), 219–229. https://doi.org/10/btk6k4. 15839499 10.1007/s10802-005-1829-8

[R7] Duncan L, Shen H, Gelaye B, Meijsen J, Ressler K, Feldman M, … Domingue B (2019). Analysis of polygenic risk score usage and performance in diverse human populations. Nature Communications, 10(1), 3328. 10.1038/s41467-019-11112-0. PMC6658471 31346163

[R8] Edenberg HJ (2002). The collaborative study on the genetics of alcoholism: An update. Alcohol Research & Health, 26, 214–218. 12875050 PMC6683843

[R9] Eme R (2016). Sex differences in the prevalence and expression of externalizing behavior. In Beauchaine TP, & Hinshaw SP (Eds.), Oxford library of psychology. The Oxford handbook of externalizing spectrum disorders (pp. 239–263). New York, NY, US: Oxford University Press.

[R10] Euser AS, Arends LR, Evans BE, Greaves-Lord K, Huizink AC, & Franken IHA (2012). The P300 event-related brain potential as a neurobiological endophenotype for substance use disorders: A meta-analytic investigation. Neuroscience & Biobehavioral Reviews, 36(1), 572–603. 10.1016/j.neubiorev.2011.09.002. 21964481

[R11] Ge T, Chen CY, Ni Y, Feng YCA, & Smoller JW (2019). Polygenic prediction via Bayesian regression and continuous shrinkage priors. Nature Communications, 10(1), 1776. 10.1038/s41467-019-09718-5 PMC6467998 30992449

[R12] Gilmore CS, Malone SM, Bernat EM, & Iacono WG (2010). Relationship between the P3 event-related potential, its associated time-frequency components, and externalizing psychopathology. Psychophysiology, 47(1), 123–132. 10.1111/j.1469-8986.2009.00876.x. 19674392 PMC2860032

[R13] Grotzinger AD, Rhemtulla M, de Vlaming R, Ritchie SJ, Mallard TT, Hill WD, … Tucker-Drob EM (2019). Genomic structural equation modelling provides insights into the multivariate genetic architecture of complex traits. Nature Human Behaviour, 3(5), 513–525. 10.1038/s41562-019-0566-x. PMC6520146 30962613

[R14] Harper J, Liu M, Malone SM, McGue M, Iacono WG, & Vrieze SI (2021). Using multivariate endophenotypes to identify psychophysiological mechanisms associated with polygenic scores for substance use, schizophrenia, and education attainment. Psychological Medicine, 52, 1–11. 10.1017/S0033291721000763. PMC8448784 33731234

[R15] Hesselbrock M, Easton C, Bucholz KK, Schuckit M, & Hesselbrock V (1999). A validity study of the SSAGA-a comparison with the SCAN. Addiction, 94(9), 1361–1370. 10615721 10.1046/j.1360-0443.1999.94913618.x

[R16] Hicks BM, Bernat E, Malone SM, Iacono WG, Patrick CJ, Krueger RF, & Mcgue M (2007). Genes mediate the association between P3 amplitude and externalizing disorders. Psychophysiology, 44(1), 98–105. 17241145 10.1111/j.1469-8986.2006.00471.x PMC2365473

[R17] Iacono WG (2018). Endophenotypes in psychiatric disease: Prospects and challenges. Genome Medicine, 10(1), 11. 10.1186/s13073-018-0526-5. 29471866 PMC5824588

[R18] Iacono WG, & Malone SM (2011). Developmental endophenotypes: Indexing genetic risk for substance abuse with the P300 brain event-related potential. Child Development Perspectives, 5(4), 239–247. 22247735 10.1111/j.1750-8606.2011.00205.x PMC3254094

[R19] Iacono WG, Malone SM, & McGue M (2003). Substance use disorders, externalizing psychopathology, and P300 event-related potential amplitude. International Journal of Psychophysiology, 48(2), 147–178. 12763572 10.1016/s0167-8760(03)00052-7

[R20] Iacono WG, Malone SM, & Vrieze SI (2017). Endophenotype best practices. International Journal of Psychophysiology, 111, 115–144. 10.1016/j.ijpsycho.2016.07.516. 27473600 PMC5219856

[R21] Karlsson Linnér R, Mallard TT, Barr PB, Sanchez-Roige S, Madole JW, Driver MN, … Dick DM (2021). Multivariate analysis of 1.5 million people identifies genetic associations with traits related to self-regulation and addiction. Nature Neuroscience, 24(10), 1367–1376. 10.1038/s41593-021-00908-3. 34446935 PMC8484054

[R22] Katsanis J, Iacono WG, Mcgue MK, & Carlson SR (1997). P300 event-related potential heritability in monozygotic and dizygotic twins. Psychophysiology, 34(1), 47–58. 9009808 10.1111/j.1469-8986.1997.tb02415.x

[R23] Keller MC (2014). Gene×environment interaction studies have not properly controlled for potential confounders: The problem and the (simple) solution. Biological Psychiatry, 75(1), 18–24. 10.1016/j.biopsych.2013.09.006. 24135711 PMC3859520

[R24] Kotov R, Krueger RF, Watson D, Achenbach TM, Althoff RR, Bagby RM, … Zimmerman M (2017). The Hierarchical Taxonomy of Psychopathology (HiTOP): A dimensional alternative to traditional nosologies. Journal of Abnormal Psychology, 126(4), 454–477. 10.1037/abn0000258. 28333488

[R25] Krueger RF, Hobbs KA, Conway CC, Dick DM, Dretsch MN, & Eaton NR, … HiTOP Utility Workgroup. (2021). Validity and utility of Hierarchical Taxonomy of Psychopathology (HiTOP): II. Externalizing superspectrum. World Psychiatry, 20(2), 171–193. 10.1002/wps.20844. 34002506 PMC8129870

[R26] Krueger RF, Markon KE, Patrick CJ, Benning SD, & Kramer MD (2007). Linking antisocial behavior, substance use, and personality: An integrative quantitative model of the adult externalizing spectrum. Journal of Abnormal Psychology, 116(4), 645–666. 10.1037/0021-843X.116.4.645. 18020714 PMC2242625

[R27] Kuo SI-C, Salvatore JE, Barr PB, Aliev F, Anokhin A, & Bucholz KK, … Externalizing Consortium. (2021). Mapping pathways by which genetic risk influences adolescent externalizing behavior: The interplay between externalizing polygenic risk scores, parental knowledge, and peer substance use. Behavior Genetics, 51(5), 543–558. 10.1007/s10519-021-10067-7. 34117972 PMC8403154

[R28] Lai D, Wetherill L, Bertelsen S, Carey CE, Kamarajan C, Kapoor M, … Foroud T (2019). Genome-wide association studies of alcohol dependence, DSM-IV criterion count and individual criteria. Genes, Brain and Behavior, 18(6), e12579. 10.1111/gbb.12579. 31090166 PMC6612573

[R29] Li C-H (2016). Confirmatory factor analysis with ordinal data: Comparing robust maximum likelihood and diagonally weighted least squares. Behavior Research Methods, 48(3), 936–949. 10.3758/s13428-015-0619-7. 26174714

[R30] Liu M, Malone SM, Vaidyanathan U, Keller MC, Abecasis G, McGue M, … Vrieze SI (2017). Psychophysiological endophenotypes to characterize mechanisms of known schizophrenia genetic loci. Psychological Medicine, 47(6), 1116–1125. 10.1017/S0033291716003184. 27995817 PMC5352523

[R31] Markon KE, Chmielewski M, & Miller CJ (2011). The reliability and validity of discrete and continuous measures of psychopathology: A quantitative review. Psychological Bulletin, 137(5), 856–879. APA PsycArticles (2011-09705-001). 10.1037/a0023678. 21574681

[R32] Muthen LK, & Muthén BO (1998–2017). Mplus User’s Guide (8th Edition). Los Angeles, CA: Muthen & Muthen.

[R33] O’Connor S, Morzorati S, Christian JC, & Li TK (1994). Heritable features of the auditory oddball event-related potential: peaks, latencies, morphology and topography. Electroencephalography and Clinical Neurophysiology/Evoked Potentials Section, 92(2), 115–125. 10.1016/0168-5597(94)90052-3 7511509

[R34] Patrick CJ, Bernat EM, Malone SM, Iacono WG, Krueger RF, & McGue M (2006). P300 amplitude as an indicator of externalizing in adolescent males. Psychophysiology, 43(1), 84–92. 10.1111/j.1469-8986.2006.00376.x. 16629688 PMC2242347

[R35] Perkins ER, Latzman RD, & Patrick CJ (2020). Interfacing neural constructs with the Hierarchical Taxonomy of Psychopathology: ‘Why’ and ‘how’. Personality and Mental Health, 14(1), 106–122. 10.1002/pmh.1460. 31456351

[R36] Porjesz B, & Begleiter H (1998). Genetic basis of event-related potentials and their relationship to alcoholism and alcohol use. Journal of Clinical Neurophysiology, 15(1), 44–57. 10.1097/00004691-199801000-00006. 9502512

[R37] Porjesz B, Rangaswamy M, Kamarajan C, Jones KA, Padmanabhapillai A, & Begleiter H (2005). The utility of neurophysiological markers in the study of alcoholism. Clinical Neurophysiology, 116(5), 993–1018. 10.1016/j.clinph.2004.12.016. 15826840

[R38] R Core Team. (2021). R: A language and environment for statistical computing. Vienna, Austria: R Foundation for Statistical Computing. Retrieved from https://www.R-project.org/.

[R39] Reich T, Edenberg HJ, Goate A, Williams JT, Rice JP, Van Eerdewegh P, … Begleiter H (1998). Genome-wide search for genes affecting the risk for alcohol dependence. American Journal of Medical Genetics, 81(3), 207–215. 9603606

[R40] Shulman EP, Smith AR, Silva K, Icenogle G, Duell N, Chein J, & Steinberg L (2016). The dual systems model: Review, reappraisal, and reaffirmation. Developmental Cognitive Neuroscience, 17, 103–117. 10.1016/j.dcn.2015.12.010. 26774291 PMC6990093

[R41] Van Beijsterveldt CE, Molenaar PC, De Geus EJ, & Boomsma DI (1996). Heritability of human brain functioning as assessed by electroencephalography. American Journal of Human Genetics, 58(3), 562. 8644716 PMC1914558

[R42] Venables NC, Hicks BM, Yancey JR, Kramer MD, Nelson LD, Strickland CM, … Patrick CJ (2017). Evidence of a prominent genetic basis for associations between psychoneurometric traits and common mental disorders. International Journal of Psychophysiology, 115, 4–12. 10.1016/j.ijpsycho.2016.09.011. 27671504 PMC5364073

[R43] Waldman ID, Poore HE, van Hulle C, Rathouz PJ, & Lahey BB (2016). External validity of a hierarchical dimensional model of child and adolescent psychopathology: Tests using confirmatory factor analyses and multivariate behavior genetic analyses. Journal of Abnormal Psychology, 125, 1053–1066. 10.1037/abn0000183. 27819467 PMC6810679

[R44] Yoon HH, Iacono WG, Malone SM, & Mcgue M (2006). Using the brain P300 response to identify novel phenotypes reflecting genetic vulnerability for adolescent substance misuse. Addictive Behaviors, 31(6), 1067–1087. 16644137 10.1016/j.addbeh.2006.03.036

[R45] Young SE, Stallings MC, Corley RP, Krauter KS, & Hewitt JK (2000). Genetic and environmental influences on behavioral disinhibition. American Journal of Medical Genetics, 96(5), 684–695. 10.1002/1096-8628(20001009)96:5&lt;684::AID-AJMG16&gt;3.0.CO;2-G. 11054778

