# Supplementary material for: Examining associations between genetic and neural risk for externalizing behaviors in adolescence and early adulthood
Source: Psychol Med. Author manuscript; Available in PMC 2025 Jan 1. (PMC11010461; doi:10.1017/S0033291723001174)
Supplement: Supplementary Material [file NIHMS1973837-supplement-Supplementary_Material.docx]

**Supplemental Methods**

**Deviations from pre-registered analytic plan**

The current study was pre-registered (https://osf.io/4f5x8 ); however, over the course of analyses we found it necessary to deviate from the pre-registered analytic plan. Originally, we planned to run analyses across smaller developmental epochs instead of adolescents (12-17) and young adults (18-31). We decided to collapse the smaller groups into adolescents and young adults after determining that the sample size would be quite small for some groups, making the analyses underpowered.

*Supplemental Table 1. Descriptive statistics and standardized factor loading from confirmatory factor analysis for externalizing indicators*

| **European Ancestry**  **(12-18 years old; *N* = 890)** | | | | | **African Ancestry**  **(12-18 years old, *N* = 480)** | | | | |
| --- | --- | --- | --- | --- | --- | --- | --- | --- | --- |
| **Variable** | **M** | **SD** | **Unstd.**  **Factor Loading** | **Std.**  **Factor Loading** | **Variable** | **M** | **SD** | **Unstd.**  **Factor Loading** | **Std.**  **Factor Loading** |
| Alcohol Use | 1.55 | 2.42 | 1.40 | .54 | Alcohol Use | .87 | 1.77 | 1.40 | .75 |
| Cannabis Use | .19 | .40 | 0.74 | .74 | Cannabis Use | .25 | .44 | 0.74 | .66 |
| ODD symptoms | .74 | 1.39 | 0.85 | .55 | ODD symptoms | 1.35 | 1.77 | 0.85 | .45 |
| CD symptoms | .72 | 1.29 | 0.80 | .61 | CD symptoms | .97 | 1.43 | 0.80 | .58 |
| **European Ancestry**  **(18-32 years old; *N* = 1,961)** | | | | | **African Ancestry**  **(18-32 years old, *N* = 922)** | | | | |
| AUD symptoms | 1.47 | 2.19 | 1.10 | .52 | AUD symptoms | 0.85 | 1.73 | 1.10 | .56 |
| CUD symptoms | 1.21 | 2.28 | 1.38 | .61 | CUD symptoms | 1.47 | 2.30 | 1.38 | .60 |
| CD symptoms | 0.90 | 1.43 | 1.02 | .71 | CD symptoms | 1.12 | 1.58 | 1.02 | .64 |
| ASPD symptoms | 1.39 | 1.65 | 1.34 | .81 | ASPD symptoms | 1.96 | 1.73 | 1.34 | .76 |

Note: *M: mean; SD: standard deviation; ODD: oppositional defiant disorder; CD: conduct disorder; AUD: alcohol use disorder; CUD: cannabis use disorder; ASPD: antisocial personality disorder; Unstd.: unstandardized; Std.: standardized.*

*Supplemental Table 2. Model Fit Statistics for the Externalizing Behavior Confirmatory Factor Analysis*

| **Adolescents**  **(12-18 years old; *N* = 1,370)** | |
| --- | --- |
| CFI | .865 |
| RMSEA | .126, 95% CI [.109, .143] |
| SRMSR | .106 |
| **Young Adults**  **(18-32 years old; *N* = 2,883)** | |
| CFI | .884 |
| RMSEA | .105, 95% CI [.092, .118] |
| SRMSR | .081 |

*Note: Fit statistics from model where item loadings were constrained across ancestry groups. CFI: Comparative Fit Index; RMSEA: Root Mean Square Error of Approximation; SRMSR: Standardized Root Mean Square Residual.*

*Supplemental Table 3. Regression results using externalizing behavior score as the criterion and including sex covariate interactions terms*

|  | **European Ancestry**  **12-18 years old** | | | **African Ancestry**  **12-18 years old** | | | | |  |
| --- | --- | --- | --- | --- | --- | --- | --- | --- | --- |
| **Predictor** | ***β*** | **[95% CI]** | **Model Fit**  **[95% CI]** | ***β*** | | **[95% CI]** | | **Model Fit**  **[95% CI]** | |
| EXT PGS | 0.10* | [0.03, 0.17] |  | -0.01 | | [-0.11, 0.09] | |  | |
| Age | 0.20* | [0.15, 0.26] |  | 0.25** | | [0.17, 0.32] | |  | |
| Sex | -0.11* | [-0.18, -0.04] |  | -0.12** | | [-0.21, -0.03] | |  | |
| EXT PGS * Sex | -0.00 | [-0.07, 0.07] |  | 0.01 | | [-0.10, 0.13] | |  | |
| EXT PGS * Age | 0.05 | [-0.01, 0.10] |  | -0.01 | | [-0.09, 0.07] | |  | |
| Age * Sex | -0.02 | [-0.08, 0.03] |  | -0.03 | | [-0.09, 0.03] | |  | |
|  |  |  | *R^2^*  = .064** |  | |  | | *R^2^*  = .075** | |
|  |  |  | [.03,.09] |  | |  | | [.03,.11] | |
| P3 Amplitude | -0.06 | [-0.13, 0.02] |  | 0.02 | | [-0.07, 0.10] | |  | |
| Age | 0.19** | [0.14, 0.25] |  | 0.25** | | [0.17, 0.32] | |  | |
| Sex | -0.11** | [-0.18, -0.05] |  | -0.12* | | [-0.21, -0.03] | |  | |
| P3 Amplitude * Sex | -0.02 | [-0.10, 0.06] |  | -0.02 | | [-0.11, 0.06] | |  | |
|  |  |  | *R^2^*  = .055** |  | |  | | *R^2^*  = .074 | |
|  |  |  | [.03,.08] |  | |  | | [.03,.12] | |
|  | **European Ancestry**  **18-32 years old** | | | **African Ancestry**  **18-32 years old** | | | | |  |
| **Predictor** | ***β*** | **[95% CI]** | **Model Fit**  **[95% CI]** | ***β*** | **[95% CI]** | | **Model Fit**  **[95% CI]** | |  |
| EXT PGS | 0.09** | [0.04, 0.13] |  | 0.14* | [0.06, 0.23] | |  | |  |
| Age | 0.11** | [0.06, 0.16] |  | 0.08** | [0.03, 0.14] | |  | |  |
| Sex | -0.26** | [-0.30, -0.22] |  | -0.32** | [-0.38, -0.25] | |  | |  |
| EXT PGS * Sex | -0.05* | [-0.10, -0.01] |  | -0.05 | [-0.14, 0.01] | |  | |  |
| EXT PGS * Age | -0.01 | [-0.06, 0.04] |  | 0.01 | [-0.05, 0.08] | |  | |  |
| Age * Sex | -0.04* | [-0.09, -0.01] |  | -0.06 | [-0.12, 0.02] | |  | |  |
|  |  |  | *R^2^*  = .089** |  |  | | *R^2^*  = .124** | |  |
|  |  |  | [.06,.11] |  |  | | [.08,.16] | |  |
| P3 Amplitude | -0.05* | [-0.09, -0.01] |  | -0.01 | [-0.07, 0.05] | |  | |  |
| Age | 0.10** | [0.05, 0.15] |  | 0.09** | [0.03, 0.15] | |  | |  |
| Sex | -0.26** | [-0.30, -0.21] |  | -0.31** | [-0.38, -0.25] | |  | |  |
| P3 Amplitude * Sex | 0.02 | [-0.02, 0.06] |  | 0.05 | [-0.01, 0.11] | |  | |  |
|  |  |  | *R^2^*  = .079** |  |  | | *R^2^*  = .107** | |  |
|  |  |  | [.06,.10] |  |  | | [.07,.14] | |  |

*Note.* Externalizing behavior score derived from confirmatory factor analysis for adolescents (comprised of alcohol use, cannabis use, DSM-5 symptom counts of Conduct Disorder and Oppositional Defiant Disorder) and young adults (comprised of DSM-5 symptom counts of alcohol use disorder, cannabis use disorder, adult antisocial personality disorder, and conduct disorder). *β* indicates the standardized regression weights. 95% CI indicates the lower and upper limits of the 95% confidence interval, respectively. Top 10 ancestry components have been regressed out of EXT PGS. * indicates *p* < .05. ** indicates *p* < .01.

*Supplemental Table 4.* *Regression results using P3 amplitude as the criterion and including covariate interactions terms*

|  | **European Ancestry**  **12-18 years old** | | | **African Ancestry**  **12-18 years old** | | |  |
| --- | --- | --- | --- | --- | --- | --- | --- |
| **Predictor** | ***β*** | **[95% CI]** | **Model Fit**  **[95% CI]** | ***β*** | **[95% CI]** | **Model Fit**  **[95% CI]** | |
| EXT PGS | -0.04 | [-0.11, 0.03] |  | 0.00 | [-0.12, 0.11] |  | |
| Age | -0.06 | [-0.13, 0.00] |  | -0.04 | [-0.14, 0.06] |  | |
| Sex | 0.04 | [-0.03, 0.11] |  | 0.06 | [-0.05, 0.14] |  | |
| EXT PGS * Sex | -0.04 | [-0.11, 0.03] |  | -0.07 | [-0.18, 0.01] |  | |
| EXT PGS * Age | -0.02 | [-0.10, 0.05] |  | 0.03 | [-0.05, 0.13] |  | |
| Age * Sex | -0.02 | [-0.09, 0.05] |  | 0.07 | [-0.02, 0.16] |  | |
|  |  |  | *R^2^*  = .010 |  |  | *R^2^*  = .015 | |
|  |  |  | [.00,.02] |  |  | [.00,.03] | |
|  | **European Ancestry**  **18-32 years old** | | | **African Ancestry**  **18-32 years old** | | |  |
| **Predictor** | ***β*** | **[95% CI]** | **Model Fit**  **[95% CI]** | ***β*** | **[95% CI]** | **Model Fit**  **[95% CI]** | |
| EXT PGS | -0.04 | [-0.08, 0.01] |  | -0.01 | [-0.09, 0.08] |  | |
| Age | -0.24** | [-0.28, -0.20] |  | -0.17** | [-0.23, -0.10] |  | |
| Sex | 0.09** | [0.05, 0.13] |  | 0.17** | [0.11, 0.24] |  | |
| EXT PGS * Sex | -0.02 | [-0.07, 0.02] |  | -0.03 | [-0.13, 0.05] |  | |
| EXT PGS * Age | 0.00 | [-0.04, 0.04] |  | 0.03 | [-0.03, 0.11] |  | |
| Age * Sex | 0.00 | [-0.04, 0.05] |  | 0.00 | [-0.06, 0.06] |  | |
|  |  |  | *R^2^*  = .068** |  |  | *R^2^*  = .062** | |
|  |  |  | [.05,.09] |  |  | [.03,.09] | |

*Note.* *β* indicates the standardized regression weights. 95% CI indicates the lower and upper limits of the 95% confidence interval, respectively. Top 10 ancestry components have been regressed out of EXT PGS. * indicates *p* < .05. ** indicates *p* < .01.
